# Seeing Global Motion in a Random Dot Image Sequence

**DOI:** 10.1177/2041669520961104

**Published:** 2020-09-30

**Authors:** Chien-Chung Chen, Hiroshi Ashida, Xirui Yang, Pei-Yin Chen

**Affiliations:** Department of Psychology, 33561 National Taiwan University, Taipei, Taiwan;; Neurobiology and Cognitive Science Center, 33561 National Taiwan University, Taipei, Taiwan; Department of Psychology, Kyoto University, Kyoto, Japan; Department of Psychology, 33561 National Taiwan University, Taipei, Taiwan

**Keywords:** coherence, illusion, aperture, random dot kinematogram

## Abstract

In a stimulus with multiple moving elements, an observer may perceive that the whole stimulus moves in unison if (a) one can associate an element in one frame with one in the next (correspondence) and (b) a sufficient proportion of correspondences signal a similar motion direction (coherence). We tested the necessity of these two conditions by asking the participants to rate the perceived intensity of linear, concentric, and radial motions for three types of stimuli: (a) *random walk motion*, in which the direction of each dot was randomly determined for each frame, (b) *random image sequence*, which was a set of uncorrelated random dot images presented in sequence, and (c) *global motion*, in which 35% of dots moved coherently. The participants perceived global motion not only in the global motion conditions but also in the random image sequences, though not in random walk motion. The type of perceived motion in the random image sequences depends on the spatial context of the stimuli. Thus, although there is neither a fixed correspondence across different frames nor a coherent motion direction, observers can still perceive global motion in the random image sequence. This result cannot be explained by motion energy or local aperture border effects.

The visual system can integrate image elements with similar spatiotemporal properties to produce a percept of global motion. A common example used in vision research is a dynamic random dot kinematogram (Braddick, 1974; Newsome & Pare, 1988; Williams & Sekuler, 1984), which contains randomly distributed dots that would shift in position from one frame to the next to create an impression of motion. If there is a sufficient proportion of dots moving in the same direction and velocity, an observer could have the impression that the whole stimulus is moving in unison.

It is commonly accepted (Sekuler et al., 1990) that two conditions have to be satisfied for an observer to perceive a coherent global motion in a multiple-element display. The first is correspondence, or that an image element in one frame should be associated with one in the next frame. When the correspondence is established, one can calculate the amount of motion, or motion energy, of the local elements. The second condition is coherence, or that a sufficient proportion of image elements should move with a direction and velocity following a specific rule. If this proportion of image elements, or coherence level, is less than a threshold, the observer would not be able to perceive a global motion.

Both correspondence and coherence are considered to be necessary for a global motion percept (Sekuler et al., 1990). Just satisfying one of these two would not produce a global motion percept. For instance, one can generate an image of randomly distributed dots and have them move by a predesignated short distance from frame to frame but with the motion direction of each dot determined randomly (see Figure 1A). If the dot distribution is sparse enough, it is not difficult to find the correct solution to the correspondence problem in this stimulus, and thus, each dot would have a constant speed (Figure 1B). On the other hand, the random walk motion direction produces zero coherence among image elements. Thus, in such random walk motion stimuli, due to a lack of coherence, one can clearly see the local motion of each dot from the frame-by-frame correspondence but have no percept of global motion.

**Figure 1.**
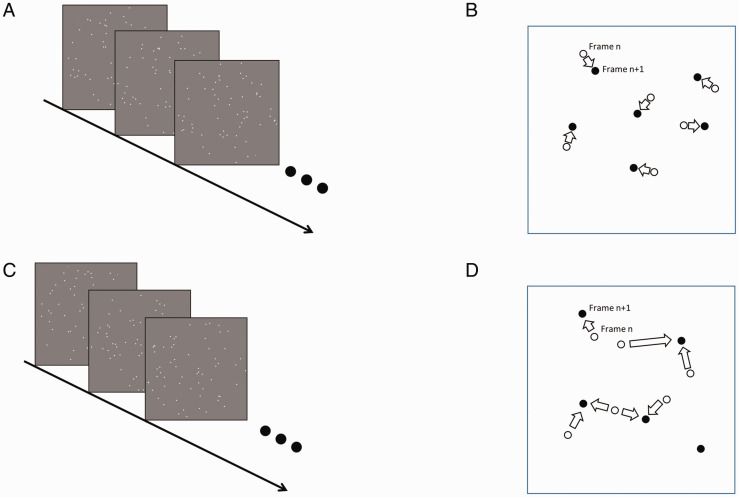
Two Types of Noncoherent Motion Are Discussed in This Article. The first is the random walk motion (A), in which all randomly distributed dots move by a predesignated distance from frame to frame but with the motion direction determined randomly for each frame. As illustrated in (B), it is not difficult for an observer to find correspondence for each dot. Thus, each dot has a constant speed albeit a different direction. The second type is the random image sequence (C), in which every frame is generated independently. There is no particular relationship between the dots from one frame to the next. Each dot in one frame can be associated with any dot in any direction and distance in the next frame (D).

Here, we demonstrate that one can perceive global motion in a stimulus containing no obvious ways to associate image elements across frames or any coherence in motion among image elements. Such a stimulus can be constructed in a very simple way as shown in Figure 1C. Each frame contains dots that are evenly and randomly distributed within a circular window. Every frame is created independently. That is, this stimulus is simply a set of random dot images presented in a sequence. Thus, because there is no particular relationship between the dots, each dot in one frame can be associated with any dot in any direction and a wide range of distances in the next frame. Such random image sequence stimuli lack not only coherence in the motion direction, as in the random walk motion stimulus discussed earlier, but also coherence in velocity (Figure 1D). One would not expect to perceive a global motion in this type of stimuli. However, as demonstrated in Supplementary Material 1, one can clearly perceive a global rotational or concentric motion in the stimuli. Here, we conducted an experiment to evaluate this effect. To capture the percept that could be missed by conventional forced-choice methods, we used a unique method of asking observers to report three types of global motion percepts for each stimulus.

## Method

### Apparatus

The stimuli were displayed on a SONY 19′ CRT by a monitor with 1,024 (H) × 768 (V) resolution, controlled by a Mac Pro computer. The visible area was 35 (H) × 26 (V) cm. At the viewing distance of 58 cm, there were 30 pixels in a one-degree visual angle. The refresh rate of the monitors was 100 Hz. The experiment control and visual stimulus generation was done in a MATLAB environment with Psychtoolbox-3 (Brainard, 1997).

### Stimuli

The stimuli were random dot kinematograms that consisted of bright dots (peak luminance 76.1 cd/m^2^) randomly distributed on a dark background (5.1 cd/m^2^) within either a circular (6.65° radius) or a rectangular (13.3° by 13.3°) window. Each dot has a spatial profile of exp(–(x–u_x_)/σ)^4^) × exp(–(y–u_y_)/σ)^4^), where (u_x_, u_y_) was the center of the dot, and the space constant, σ, was 2’. There were 80 dots in each frame. The stimuli were refreshed every 40 ms or four video frames. The duration of the stimulus was 800 ms.

There were three types of stimuli used in this experiment: The first was *random walk motion*, in which each dot was displaced by 20′ between each refreshment, giving each dot a velocity of 8.3 degree/s. The motion direction of each dot was randomly determined frame by frame. The second stimulus type was a *random image sequence*, also called random position noise in some literature (Scase, Braddick & Raymond, 1996). That is, in each frame, all of the dots were generated independently from other frames and other dots. One such stimulus was practically a set of independent random dot images presented in a sequence. The third type, used for catch trials, was *global motion stimuli*, in which 35% of dots moved with a predesignated direction, whereas the other 65% of dots each moved with a random determined direction. This configuration allowed an observer to perceive a motion of a global pattern. There were three types of global motions: radial, concentric, and linear. Each stimulus presentation lasted 4 seconds. A video file showing examples of the random image sequences can be seen in Supplementary Materials.

### Procedure

Each trial started with a uniform gray with a fixation mark at the center of the display, followed by the stimulus presentation for 800 ms. After the stimulus presentation, the participants were asked to rate the strength of the perceived global motion for all three types (linear, concentric, and radial) consecutively in a randomized order when they were cued by a question displayed on the screen. The response was made on a 7-point Likert scale, with 1 being *no global motion of the prompted type*, and with 7 being the *strongest and most exclusive of other types*. The order of the three types of responses was randomized for each trial.

Before the start of the data collection, the participants were given 20 practice trials to familiarize themselves with the tasks and to establish criteria for motion judgment. For each participant, there were 20 trials for the random image sequence condition and 20 for the random walk motion conditions. These 40 trials, which contained no global motion information by themselves, was balanced by another 40 trials meant to create a percept of global motion. They included 20 for linear global motion (5 each for up, down, left, and right motions, respectively), 10 for concentric motion (5 each for clockwise and counterclockwise conditions, respectively), and 10 for radial motion (5 each for expansion and contraction conditions, respectively). The order of all 80 stimuli was randomized for each participant.

There were 12 participants involved in this study. All of them had normal or corrected-to-normal visual acuity (20/20) at the time of the experiment and no known history of neurological disorder. The participants gave written consent to participate in this experiment. The procedure was approved by the ethics committee of the Unit for Advanced Study of Mind, Kyoto University.

## Results

**Figure 2.**
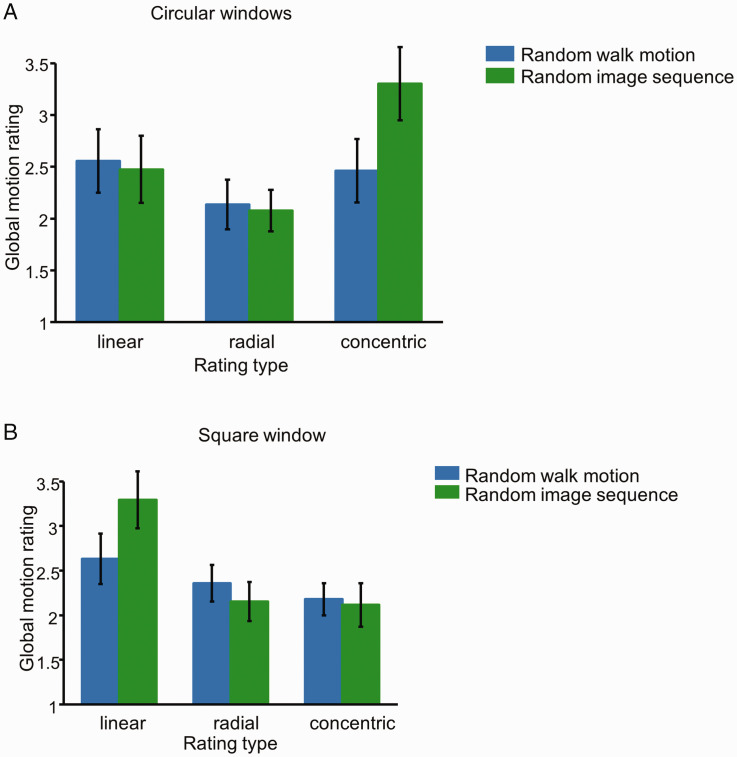
Rated strength of linear, radial, and concentric global motions through a circular (A) or rectangular (B) viewing window. In each panel, the blue bars are for a random walk motion, whereas the bars are for a random image sequence. For a comparison, the 35% coherent motion stimuli were rated 5.3 to 5.7 for their corresponding motion type and 1.7 to 2.4 for other type.

Figure 2 shows the perceived global motion intensity, as rated by the participants, of both the random walk motion and the random image sequence in three criteria. In the random walk motion stimuli, not surprisingly, the participants hardly perceived any global motion: They gave only weak intensity ratings on all types of global motions (2.1–2.5) for those stimuli. On the other hand, the participants were able to perceive global motion in the random image sequence. As shown in Figure 2A, the participants rated concentric motion stronger than either linear (repeat-measure *t* test, *t*(11)=2.66; *p* =.011, or radial motion, *t*(11)=3.82, *p*=.0014, in the random image sequence stimuli. Furthermore, although the rated perceived concentric motion (3.3) was weaker than that for the real motion stimuli (5.4), it was significantly stronger than that in the random walk motion stimuli, *t*(11)=5.42, *p* <.001. Meanwhile, there was no statistically significant difference in either the linear or the radial motion rating between the two types of stimuli.

Such global motion seems to partially depend on the global context. As shown in Figure 2B, when the stimuli were viewed through a square window instead of a circular one, the participants rated linear motion stronger in the random image sequence than either concentric (repeat-measure *t* test, *t*(11)=2.47; *p* =.016, or radial motion, *t*(11)=3.02, *p*=.006, in the random image sequence. The participants showed no such bias in random walk motion stimuli. Furthermore, the perceived linear motion in the random image sequence was stronger than that in the random walk motion stimuli, *t*(11)=3.28, *p*=.0037. Meanwhile, there was no statistically significant difference in either the concentric or the radial motion rating between the two types of stimuli.

These results can be confirmed by a repeat-measure three-way factorial (2 stimulus type × 3 rating criteria × 2 window shape) analysis of variance, which showed a significant rating criteria main effect, *F*(2, 121)= 5.75, *p*=.0047, the two-way interaction between the rating criteria and window shape, *F*(2, 121)=6.88, *p*=.0015, and the three-way interaction of all within-subject factors, *F*(2, 121)=3.09, *p*=.048. The subsequent simple-interaction analysis of random walk motion conditions showed no significant main effect or interaction, suggesting that neither the rating criterion nor the window shape affected the participants’ ratings for such stimuli. This is consistent with the notion that the participants perceived no global motion for random walk motion stimuli. On the other hand, the simple-interaction analysis of the random image sequence conditions showed a significant rating criterion main effect, *F*(2, 212)=5.86, *p* = .0033, and the interaction between the rating criteria and window shape, *F*(2, 212,)=9.27, *p* = .0001. This result is consistent with the a priori *t*-test analysis that the participant tends to perceive concentric global motion in the random image sequence with a circular window and linear global motion with a square window.

## Discussion

**Figure 3.**
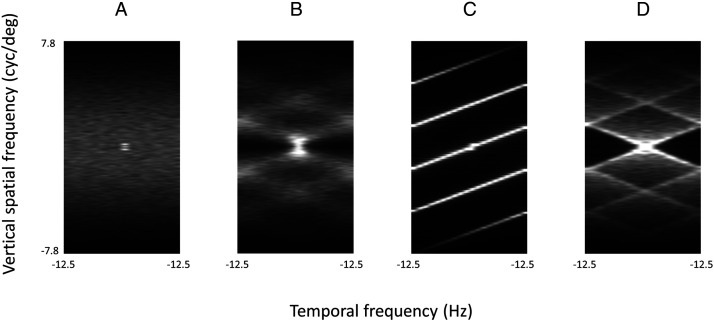
The spatiotemporal power spectrum of (A) random image sequence, (B) random walk motion, (C) 35% coherent vertical linear motion, and (D) 35% coherent concentric motion. Each panel showed the spectrum averaged across 40 stimuli of the same type used in our experiment. For simplicity, here, we plot the spatial frequency spectra for vertical modulation against temporal frequency.

Here, we showed that it is possible to perceive global motion in a sequence of random dot images. This effect cannot be explained by motion energy (Adelson & Bergen, 1985). The motion of an object in a visual stimulus can be considered as a change of light intensity in both space and time. Such change, after the Fourier transform of the visual stimulus, should produce a concentration of energy in the spatiotemporal power spectrum at the location where the ratio between temporal and spatial frequencies corresponds to the velocity of movement. To test whether our effect can be accounted for by motion energy, we performed Fourier transform on our stimuli along both spatial (x and y) and the temporal (t) dimensions to get their three-dimensional spatiotemporal power spectra. We then averaged the spectra of the stimuli of the same type to obtain the spectra shown in Figure 3. For simplicity, we plot y–t, or vertical spatial frequency versus temporal frequency, spectra here.

As shown in Figure 3A, the spatiotemporal frequency spectrum for the random image sequence is quite uniform. There is no concentration of energy, except for near the DC component, throughout the spectrum. Thus, there is no bias in motion energy toward any particular type of motion. On the other hand, the spatiotemporal frequency spectrum of typical random kinematograms with linear (Figure 3C) or concentric (Figure 3D) global motion at 35% coherence, as expected, shows a distinct concentration of energy for a specific spatial-temporal frequency ratio. The spectrum for the random walk motion (Figure 3B) also showed a concentration of energy for a particular spatial to temporal frequency ratio, although it was not as highly concentrated as that of the linear motion (Figure 3C), reflecting constant displacement from one frame to the next. Thus, the perceived global motion in the random image sequence cannot result from Fourier motion energy.

The position of dots in a frame of our random image sequence stimuli was created with a random number generator. Thus, there may be a concern that our result might be affected by accidental features in the stimuli. That is, there was a possibility that the positions of certain dots in one frame, by chance, might have the same displacement from their respective corresponding dots in the previous frame. If there were enough proportion of dots with such accidental feature, the stimuli would be essentially a low-coherent real motion stimulus and thus produce a high global motion rating among the participants.

**Figure 4.**
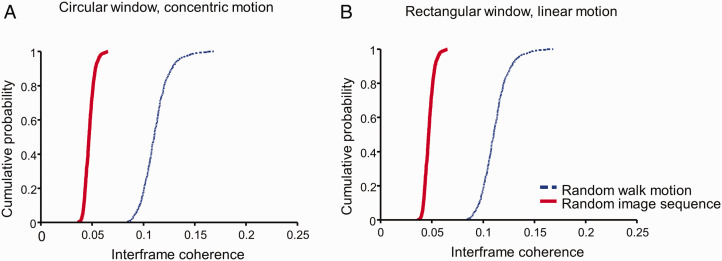
Interframe motion coherence for random walk motion (blue dashed curve) and random image sequence (red solid curve) in (A) circular window and (B) rectangular window. We computed the cross-correlation between each frame and its subsequent frame and took the max of the result. This max cross-correlation is then divided by the geometric mean of the max of the autocorrelations of the two frames to get interframe coherence. The circular windowed stimuli were morphed from Cartesian to polar representations to account for radial and concentric motion. For visualization, we sorted coherence from smallest to largest. The cumulative probability was calculated as the order of a coherence divided by the total number of coherence in the respective category.

To assess such possibility, we first examined whether there was an unusually large proportion of dots with a coherent displacement across the frames. We computed the cross-correlation between each frame in our rectangle window stimuli and its subsequent frame and took the max of the result. This is equivalent to find the greatest coherent motion at any speed and in any directions that can be produced by our stimuli. For the stimuli with circular window, we performed the same computation after first morphing the stimuli from Cartesian to polar coordinate to account for concentric and radial motion. The cross-correlation (Figure 4A and B), scaled by the geometric mean of the max autocorrelations of the two frames, was from 0 to 0.09 for the random image sequences (red solid curves in Figure 4A and B) and from 0.01 to 0.20 for random walk motion (Figure 4B). Notice that, the random image sequence has less motion coherence than random walk motion even though the former produced a stronger global motion percept (Figure 2). It is expected because a dot in a frame of a random walk motion stimulus must be placed on a 10’ radius circle around its corresponding dot in the previous frame, whereas the dot placement within a frame in a random image sequence was not constrained. Thus, there was no evidence of exceeding large coherent displacement in our random stimuli.

**Figure 5.**
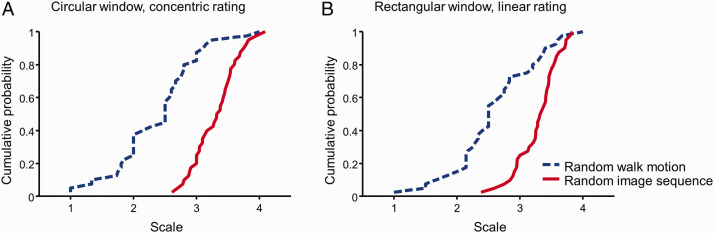
The concentric (A) and the linear (B) global motion rating for each of random walk motion (blue dashed curve) and random image sequence (blue solid curve) stimulus. For visualization, we sorted rating in each category from smallest to largest. The cumulative probability was calculated as the order of rating divided by the total number of stimulus in its category.

We then examined the distribution of the global motion rating for each individual stimulus. If our result that a participant can perceive global motion in random dot image sequences but not in random walk stimuli was due to the occurrence of accidental features, we would expect the distributions for the random image sequence and the random walk stimuli to be similar except some outliers with high rating for the random image sequence. This is inconsistent with our result (Figure 5). The two distributions were vastly different: The Kolmogorov–Smirnov test *D* = 0.7, *p* <.0001 for concentric motion rating in the circular window condition, and *D* = 0.6, *p* < .0001 for parallel motion rating in the rectangular window condition. Actually, even the lowest concentric motion rating in the random image sequence was higher than the rating of 65% of the random walk stimuli. Thus, the difference in perceived global motion between the random image sequence and random walk stimuli was systematic and not accidental.

We found that the perceived global motion in random image sequence depends on the shape of the viewing window: The participants perceived a concentric motion with a circular window, whereas they perceived a linear motion with a rectangular window. Thus, some may argue the perceived global motion may result from a bias of local motion at the edge of the window. For instance, because a dot cannot associate with anything outside of the window, it has a greater probability of associating with another dot in the direction parallel to the edge of the window than one would presume with a completely random association. Or, as demonstrated in the barberpole illusion (Badcock McKendrick & Ma-Wyatt, 2003; Guilford, 1929; Kooi, 1993; Wallach, 1935), the edge of the window provides a solution to the aperture problem and thus makes the global motion appear to be parallel to the edge. . If there are enough number dot associations parallel to the edge, the visual system may be able to pick them up and allow an observer to perceive a global motion. This interpretation, however, would encounter many difficulties. First, unlike the terminating points of the 1-D grating in the barberpole illusion, which have only one direction of motion along the edge, in the random image sequence, a dot can go in either directions along the edge individually. It is rather difficult to have enough dots moving along the same direction. Second, the largest probability bias should be in the direction away from the edge of the window. Furthermore, this bias, unlike the one parallel to the edge, is unidirectional. Hence, one should expect with a circular window to perceive a contracting radial motion in the random image sequence. Yet, our participants reported no such percept. Second, such probability bias also occurs for random walk motion stimuli. Yet, they also reported no radial global motion percept in such stimuli. Thus, the edge of the window cannot explain our result.

Hsieh and Tse (2006) refreshed a random dot pattern surrounded by a stationary random dot pattern. They reported that the observers can perceive a zipper-like motion in the refreshing part of the display. They called this phenomenon *illusory rebound motion* for the perceived motion direction after each refreshment was opposite from the previous refreshment. Notice that the stationary surround is important for Hsieh and Tse’s (2006) experiment, for they interpreted their result in terms of an illusory line motion (Hikosaka et al., 1993) in which a horizontal bar presented shortly after a reference stimulus appears to shoot away from it. That is, the stationary surround provides such a reference and, in turn, the first illusory motion. Then, the heuristic that the “visual system tends to interpret objects as moving from where they last stopped moving” (Hsieh & Tse, 2006, p. 1927) provided the subsequent illusory rebound motion. Our result is different from theirs in two major ways. First, in our display, we did not have a surround pattern. Thus, it is unlikely that the illusory line motion was involved. Second, instead of a change in the motion direction from one frame to the next, observers would perceive a coherent motion in the same direction for many frames, if not for the entire stimulus duration of a trial. Thus, our effect is unlikely to be a rebound motion from the previous frame. In addition, the perceived global motion in the random image sequence should have nothing to do with illusory line motion (Hikosaka et al., 1993) or rebound motion (Hsieh & Tse, 2006).

A similar type of stimuli to our random image sequence is the dynamic Glass pattern. A Glass pattern (Glass, 1969) is composed of randomly distributed dot pairs, or dipoles. If the orientation of the dipoles conforms to a certain geometric transform, observers perceive a global pattern in the image. Ross et al. (2000) showed that an observer can perceive global motion with a sequence of Glass patterns, and the direction is consistent with the orientation of the dipoles. The common interpretation is that the orientation of the dipoles biases the motion system and thus produces a global motion percept. However, in our random image sequence, no such local orientation exists. Thus, the orientation of local image elements, such as dipoles, may not be a necessary condition for perceived global motion.

Finally, in the circular window condition, an observer tends to perceive a concentric motion rather than a radial one, even though the image properties should either be neutral for both or favor the latter. Perhaps a concentric motion is a default mode of motion percept in the visual system, although it is subject to scene interpretation and thus can be replaced under different viewing conditions.

## Supplementary Material

Supplemental Video 1
